# Comparing Virtual Reality and Robotic Training Effects on Balance Ability and Confidence in Older Adults

**DOI:** 10.3390/app15115909

**Published:** 2025-05-24

**Authors:** Oluwasola Okhuoya, Lara A. Thompson

**Affiliations:** Center for Biomechanical & Rehabilitation Engineering, Biomedical Engineering Program, School of Engineering and Applied Sciences, University of the District of Columbia, 4200 Connecticut Ave. NW, Washington, DC 20008, USA

**Keywords:** aging, elderly, virtual reality, balance training, balance confidence, falls

## Abstract

Falls are the leading cause of injury and mortality among older adults—one in four individuals 65 years old and above experiences falls. Thus, balance training interventions that improve balance ability and reduce the risk of falls are of critical importance. Through two complementary interventions, our research sought to determine the effects of Virtual Reality (VR) compared to Robotic-Assisted Balance Training (RABT) on balance ability and balance confidence in older adults. The VR intervention utilized Oculus headsets to create immersive balance exercises, while the RABT employed a multidirectional overground robotic system (NaviGAITor). Participants (aged 60–85 years old) underwent a 6-week training protocol consisting of two 30 min sessions per week. Balance ability was quantified using center of pressure (COP) parameters and the Balance Error Scoring System (BESS), while balance confidence was measured using the Activities-Specific Balance Confidence (ABC) scale. Results indicated no statistically significant differences between the training methods. However, the RABT group showed trends toward enhanced balance performance, with observed decreases in mediolateral (ML) maximum displacement during wide stance conditions and reductions in BESS errors on both firm and foam surfaces. The VR group demonstrated significant changes in ML RMS values during tandem stance (*p* = 0.045) and improved participants’ relationship with perceived and actual balance ability (increased correlation between BESS errors and ABC scores from R^2^ = 0.00 pre-training to R^2^ = 0.65 post-training). Balance confidence did not significantly increase in either group. These findings suggest that while RABT may trend toward improvements in objective balance parameters, VR training appears to enhance participants’ perceptual accuracy of their balance capabilities.

## 1. Introduction

With the growth of the aging demographic worldwide, by 2050, the number of elderly people is expected to reach 1.5 billion, accounting for 16% of the world’s total population. Since the aging population is rapidly increasing, and imbalance, which leads to falls, is a major concern for this demographic, a specific focus on balance rehabilitation methods and technologies are critical. According to the Centers for Disease Control and Prevention, within the United States each year over twenty-five percent, or over 14 million, adults sixty-five (65) years old or older have suffered a fall; nearly 40% of these fall victims are treated for fall-related injuries [1,2]. One in ten falls results in an injury severe enough to limit daily activities or require medical attention [3]. Annually, falls among older adults account for approximately three million emergency department visits and one million hospitalizations [3]. Beyond physical harm, the fear of falling can lead to reduced physical activity, muscle weakness, and an increased risk of future falls [3]. The consequences of falls can be severe (death, injury, and hospitalization), cause increased anxiety about daily activities (leading to reduced mobility), and ultimately cause a loss of independence, affecting the quality of life. The prevalence of frailty in people aged older than 65 years is 7.2%, increases with age, and represents the main risk factor for incident falls, disability, hospitalization, and death [4]. The diagnosis of frailty comprises several domains, including weight loss, weakness, exhaustion, slowness, and low physical activity [4]. Falls are a major concern for all older adults. But aside from the loss of balance leading to the act of falling, one may possess an over-concern (fear) related to the anticipation of falling—this can ultimately limit one’s confidence and willingness to go about their daily activities [5]. Due to gait and balance deficiencies being the primary risk factors for falls in older persons, it is crucial to understand the effects of training on the stability of balance and gait [6]. Further, it is also important to examine the dynamic interaction between (actual) balance ability and balance confidence (perceived balance ability); lower perceived balance ability (or decreases in balance confidence for daily activities) can limit one’s quality of life.

Traditional rehabilitation programs, while beneficial, often face limitations in accessibility, engagement, and/or long-term adherence. As a result, emerging technologies such as robotic-assisted balance training (RABT) and Virtual Reality (VR) have gained attention as an innovative and potentially effective alternative for improving balance, mobility, and fall prevention among older adults.

### 1.1. Robotics for Balance Training

Robotic assisted balance training (RABT) employs the use of electromechanical devices that facilitate movement (e.g., during stepping cycles by supporting body weight and/or while automatizing the gait process through facilitation of movement of several lower limb joints) [7]. In other words, robotic balance training uses robotic devices to guide individuals through exercises and, in some cases, can provide feedback on their performance [8].

In terms of examples of specialized robotic systems, the ZeroG, Autoambulator, Biodex Unweighing System, and Lokomat System have been previously utilized [1]. The ZeroG involves a robotic system that supports body weight, allowing individuals to practice balance exercises in a controlled setting [1]. The goal of the ZeroG overground body-weight support system is that it aids patients with significant gait issues toward safely practicing gait and balance exercises, offering both static and dynamic support through a custom-series elastic actuator attached to a driven trolley moving on an overhead rail [9]. Even though the benefits of the ZeroG system are promising, there is a need for further studies and/or clinical trials to validate its effectiveness across different groups, including older adults. This empirical evidence would expand its generalization within rehabilitation settings and beyond. Jalal et al. (2024) highlighted that exoskeleton robots can help elderly individuals with daily tasks [10]. Various exoskeletons have been developed to aid the elderly, and those with walking impairments, as explained in the study. Robot-aided therapy, including exoskeleton therapy, has proven successful in keeping patients motivated and invested in their treatment [11]. These technologies can provide personalized and interactive rehabilitation experiences, making therapy sessions more enjoyable and engaging for individuals with upper or lower limb impairments. However, many studies did not evaluate the effectiveness of robot-assisted therapy in comparison to conventional physical therapy nor other therapies. A previous study in older adults demonstrated that training with a balance exercise assist robot is more effective than conventional training [12]. The explanation provides valuable information about the relative efficacy of robot-assisted therapy versus traditional physical therapy.

RABT has significant promise in enhancing balance and functional independence in older people. A study involving the Balance Exercise Assist Robot (BEAR) found that frail older adults who participated in robotic balance exercises exhibited increased gait speed, improved Timed Up and Go (TUG) performance, and enhanced muscle strength [12]. Importantly, participants expressed greater satisfaction and preference for robotic training over conventional methods, suggesting that robotic systems may improve engagement and adherence in rehabilitation programs. While robot-assisted gait training has shown potential in enhancing balance and confidence among older adults, there exist considerable limitations. Finding suitable participants for this type of training can be difficult, particularly among older adults who may have varying levels of mobility and health conditions [13]. High dropout rates are also a significant concern, as they can skew results and limit the effectiveness of such programs. Factors contributing to dropout include discomfort with technology, lack of perceived benefit, and long training durations which are often required to induce significant physical changes, as effective outcomes were noted in studies where interventions lasted over two months [14,15]. Thus, it is important to further explore RABT for older adults’ balance.

### 1.2. Virtual Reality (VR) and Robotic-VR Combined Balance Training

There is a need for balance training protocols that are accessible (with the potential to be transitioned to one’s home environment) and engaging (particularly for those with limited mobility or access to healthcare facilities). Virtual reality (VR)-based training programs offer a promising alternative to traditional methods [10], providing balance and mobility exercises to older adults that are accessible (i.e., able to access in one’s home), yet effective. Through immersive experiences, VR-based training programs may help older adults to practice balance and mobility exercises in a controlled setting, practiced within one’s home, with the goal being to risk of falls and injuries through training. A growing body of evidence suggests that VR-based training programs can be effective in improving balance and reducing falls, particularly when compared to traditional training methods. For example, a 2019 systematic review and meta-analysis reported that VR-based training programs were more effective than traditional training methods in improving balance and reducing falls in older adults [10].

More recent studies indicate that VR-based interventions are highly effective in enhancing balance and gait in older adults. A 2020 randomized clinical trial comparing Virtual Reality Gait Training (VRGT) to standard treadmill training found that VRGT participants exhibited significant improvements in walking velocity, step width, stride length, and step length, reinforcing VR’s potential in gait rehabilitation [16]. A 2021 study on immersive VR demonstrated that older adults with balance disorders who underwent VR-based balance training showed improved mobility and reduced dizziness symptoms compared to those receiving conventional physiotherapy. This suggests that immersive VR may provide superior benefits by addressing both sensory and mobility deficits [17]. Similarly, a 2021 randomized controlled trial examined Xbox Kinect-based VR exercises for fall-prone older adults. The results showed significant improvements in the Berg Balance Scale (BBS) and Timed Up and Go (TUG) test scores in the VR group. Furthermore, participants reported a notable reduction in the fear of falling, highlighting VR’s psychological benefits in building confidence and reducing fall-related anxiety [18].

Although recent technological advancements, such as virtual reality (VR) and robotic-assisted balance training (RABT), have emerged as innovative tools for balance rehabilitation, there is limited research directly comparing the effectiveness of these two approaches in improving balance performance. The use of exoskeletons with VR, augmented reality, and gamification for balance training in survivors of stroke could aid patients in their recovery [11]. Further, it discussed the effectiveness of robot-assisted treatment as an innovative and engaging therapy. Because traditional therapy can become monotonous and exhausting for patients, the above treatments could increase motivation and adherence. However, more research is needed, and the above study focused on a specific population (survivors of stroke) limiting the generalizability of the findings to other patient groups [11]. It is essential to assess the effectiveness of these rehabilitation techniques across a wider range of older individuals.

Here, we sought to examine if VR-based balance training or RABT has greater impacts on balance performance and perception in healthy older adults. Identifying effective methods is crucial for enhancing balance, thus reducing falls, in older adults. By investigating comparisons of innovative approaches, such as robotic and VR balance training, we aim to contribute to new knowledge which may ultimately lead to the development of more effective, personalized rehabilitation methods that can enhance mobility, independence, quality of life, and overall well-being for individuals aged 65 years old and older. While both methods have shown promise, there is currently limited research directly comparing their impact on improving balance performance in older adults and much emphasis has been on survivors of stroke. Identifying which approach is more effective can provide critical insights that enable the development of targeted, evidence-based interventions to maximize balance improvement, reduce fall risk, and better support the unique needs of the aging population. Our study holds impact in that there has not been any previous study that contrasted the effectiveness or robotic and VR training methods on balance performance and balance confidence in older individuals. Therefore, this work could bring about the development of innovative training programs that have the potential to improve the lives of individuals across a range of populations and occupational contexts. Furthermore, these interventions have broader implications for the fields of healthcare and technology, as they may lead to the development of new methodologies and technologies, as well as approaches for fall prevention and rehabilitation.

## 2. Materials and Methods

Here, we focused on two distinct balance training approaches (VR and RABT) designed for older adults. Protocols were approved by the University of the District of Columbia’s (UDC’s) Institutional Review Board (#2073871-01 and #979744-1). All participants gave their informed consent prior to taking part in the study and the research was conducted in the UDC Center for Biomechanical and Rehabilitation Engineering (CBRE).

### 2.1. Participants

Participants for both interventional groups were older adults aged 60–85 years, recruited primarily through campus postings, word of mouth, and additional outreach by UDC’s Institute of Gerontology. Inclusion criteria included that participants be able to ambulate independently for a distance of at least 10 feet and score above 25 on the Mini-Mental State Examination (MMSE). Participants in both groups predominantly scored close to 30 (the perfect score) on the MMSE, with no individual scoring below 28. The VR-trained group included 9 participants who completed the VR intervention (mean age 75.9 ± 3.7 years). The robotic-trained (RABT) group included 16 healthy individuals (69.7 ± 6.7).

### 2.2. Training

**Figure 1.**
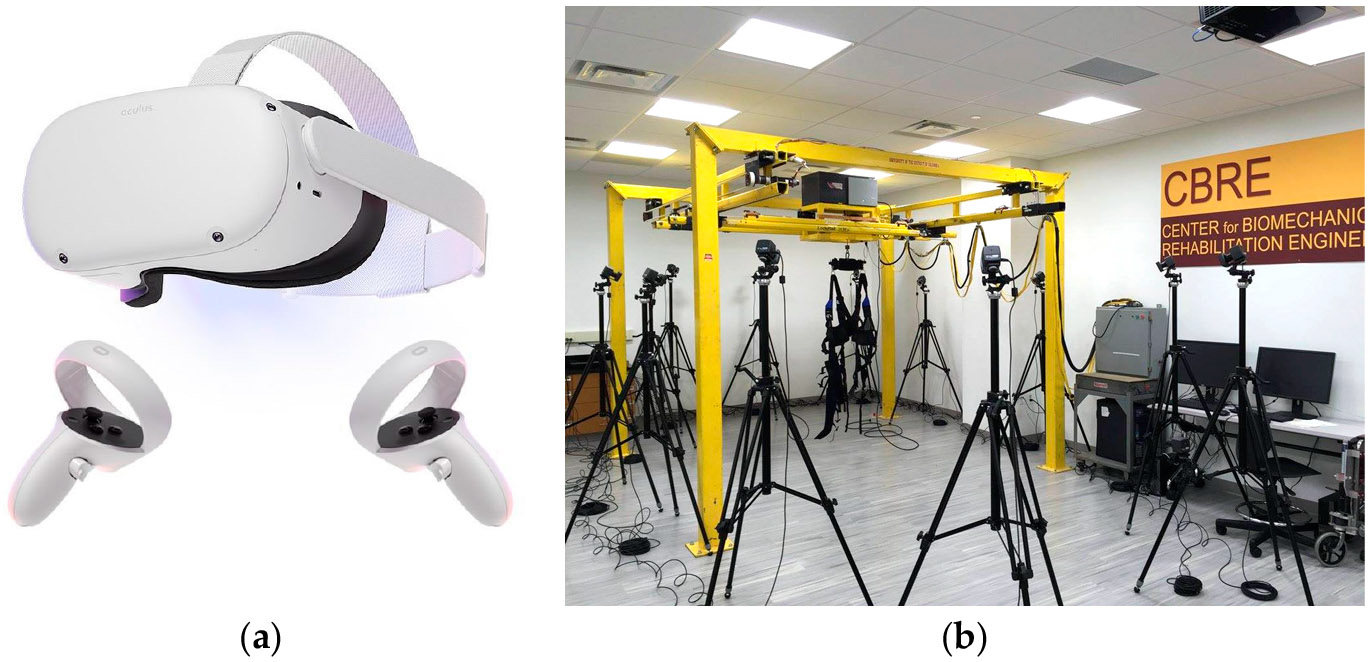
(**a**) VR training headsets and setup for (**b**) RABT with the NaviGAITor device.

The VR-based training study utilized Oculus headsets (Figure 1a) to engage participants in interactive balance exercises, while the RABT (Figure 1b) employed multidirectional overground robotic systems designed to enhance stability. Both training programs spanned six weeks, consisting of two 30 min sessions per week, totaling 12 sessions. The VR group utilized a static virtual environment while undergoing training. In contrast, the RABT group required participants to perform exercises with a multidirectional robotic system (NaviGAITor) while viewing the regular laboratory environment; the robotic training was non-bodyweight supportive and directly followed the movements of the participant; however, the system provided light resistance as the subject walked. All groups underwent training exercises and assessments barefoot to optimize somatosensory feedback.

The training difficulty increased through adjustments to the complexity of balancing tasks and by modifying sensory inputs and the base of support [19]. During the first two weeks, participants began with fundamental walking exercises that included both forward and backward movement, incorporating wide and tandem stepping patterns. As participants moved into weeks three and four, the difficulty increased with the introduction of foam surfaces that varied in density. This phase included standing balance exercises, targeted leg movements, squats, and walking across unstable surfaces. The final two weeks combined all previous elements into more complex challenges, such as navigating obstacles while on foam surfaces, with deliberate manipulation of sensory inputs. The only difference was that we could not perform gait training exercises or VR training. To evaluate the effectiveness of the interventions, each study employed compared pre-intervention and post-intervention balance performance metrics. Safety was a priority in both studies, with individual training sessions supervised by research assistants and principal investigators. Participants were monitored closely, and safety protocols were followed to ensure their well-being throughout the training period.

### 2.3. Balance Assessment

Balance is frequently investigated to assess the recovery of patients who have suffered from trauma or disease [6]. Multiple approaches to assess gait, balance, and functional mobility have been developed including the Timed Up and Go (TUG) test, FES (Falls Efficacy Scale), ABC (Activities-specific Balance Confidence Scale), BESS (Balance Error Scoring System) [19]. To ensure static stability during the process of locomotion, one’s center of mass must be vertically projected inside the polygon of support formed by the contact points between its limbs and the ground, or base of support [4]. Quiet (i.e., static) standing is an assessment of balance performance, representing an individuals’ ability to constrain the center of mass (COM) or center of pressure (COP) within an established base of support (BoS) [2]. The COP trajectory is recorded using force platforms, which track the point of application of the ground reaction forces resultant under the feet. The COP represents the point of application of the ground reaction force vector and provides insight into postural stability by examining the displacement and variability of body sway. The resulting signal, called stabilogram, is frequently analyzed using either its one-dimensional variations in the mediolateral (ML) or anteroposterior (AP) direction, or its two-dimensional trajectory [3].

Here, to assess balance performance in older adults, pre- and post-training evaluations were conducted for both the VR and RABT interventions, balance performance was assessed using the Balance Error Scoring System (BESS) and center of pressure (COP) metrics were obtained from force plate data. BESS assessment was used as a clinical measure for balance which allows for variation in somatosensory cues (i.e., hard vs. foam support surface) as well as variation in base of support, BOS, (stance widths of wide, tandem, and single-leg) while the participant has no visual cues (eyes are closed), thereby allowing for measured differences in balance (in terms of BESS errors) with increases in task difficulty for six stance conditions [19]. Participants were instructed to stand upright with their eyes closed and hands on their hips for 20 s per condition. During each stance, observable deviations from the instructed posture, referred to as “errors”, were recorded. Errors included actions such as extending the arms away from the hips, opening the eyes, stepping or stumbling, excessive hip flexion (>30°), or losing balance for more than five seconds. An evaluator scores the subject’s postural stability using an objective list of errors. Each error committed is given 1 point, and the maximum allowable number of points for each position is 10. The subject’s total BESS score is the sum of the individual stance position scores (maximum total BESS score is 60) [4]. Higher scores indicated greater postural instability, whereas lower scores reflected better balance control. The COP assessment was performed to examine postural control during balance tasks. Force plate data were collected using the Tekscan Strideway system (Tekscan Version 7.8, Norwood, MA) at a sampling frequency of 50 Hz during five 20 s trials per stance condition. Participants completed double-leg, single-leg, and tandem stances with eyes open and closed.

In order to assess balance confidence, the ABC scale was used. The ABC scale is a self-reporting assessment designed to gauge an individual’s confidence in their ability to carry out different daily living tasks without losing their balance. Balance confidence levels were reported by the participant for daily living scenarios in and out of one’s home, using scores ranging from 0% (no confidence) to 100% (complete confidence). All ABC assessments were administered in person by trained research assistants. The researcher verbally guided each participant through the individual questions and scenarios, ensuring comprehension of each item before the participant provided their confidence rating. This in-person assessment ensured consistency in how participants interpreted the scenarios and reported their self-perceived balance confidence across both intervention groups.

### 2.4. Data Analysis

The COP data were post-processed using MATLAB software (MATLAB R2023b, Natick, MA, USA). Key COP metrics, including anterior–posterior (AP) and medial-lateral (ML) displacement, were calculated from the collected data. From the COP data, features (root mean square (RMS) and peak-to-peak displacement (MAXD)) were extracted and are described below in Equations (1) to (6).

AP COP time series without offset:

(1) $$\text{AP}=\text{APo}-\frac{1}{N}\sum |AP[n]|$$

ML COP time series without offset:

(2) $$\text{ML}=\text{MLo}-\frac{1}{N}\sum |ML[n]|$$

where APo∕MLo:AP∕MLCOP path relative to the origin of the force platform coordinate system AP∕ML and N=number of data points.

AP Root Mean Square (${\text{RMS}}_{\text{AP}}$):

(3) $$RMS_{AP}={\left[\frac{1}{N}\sum \text{AP}{\left[n\right]}^{2}\right]}^{1∕2}$$

ML Room Mean Square (${\text{RMS}}_{\text{ML}}$):

(4) $$RMS_{ML}={\left[\frac{1}{N}\sum \text{ML}{\left[n\right]}^{2}\right]}^{1∕2}$$

where

N is the total number of data points,

∑ represents the summation over all data points.

AP Peak-to-peak Displacement (${\text{MAXD}}_{\text{AP}}$):

(5) $${\text{MAXD}}_{\text{AP}}=\max(\text{AP})-\text{min}(\text{AP})$$

ML Peak-to-peak Displacement (${\text{MAXD}}_{\text{ML}}$):

(6) $${\text{MAXD}}_{\text{ML}}=\max(\text{ML})-\text{min}(\text{ML})$$

These COP metrics provided quantitative measures of postural stability and balance control, facilitating comparisons between pre- and post-training performance.

After pre-processing in MATLAB, the RABT participants’ data were tabulated and post-processed using Microsoft Excel (Version 16.26). Pre- and post-training trials were pooled for each test condition, from which mean and standard error of the mean (SEM) were computed. In Excel, BESS errors were analyzed by calculating the mean errors across stance conditions to assess deviations in balance performance. This approach aimed to determine whether stance deviations (BESS errors) changed as a function of increasing task difficulty (BESS test conditions) when comparing post- to pre-training results. The BESS errors were calculated both for individual stance conditions and as a total across all conditions. For each intervention, pre- and post-training averages (means) and SEMs were computed across trials for multiple balance performance parameters, including BESS errors, center of pressure (COP) metrics related to displacement.

In terms of statistical analysis, the data for both the RABT and VR balance training interventions were systematically organized, processed, and analyzed to evaluate changes in balance performance among older adults following training. The statistical analysis was conducted using MATLAB (MATLAB R2023b, Natick, MA). To evaluate the VR-based and RABT interventions, changes in balance performance metrics (defined above) were analyzed for both groups. A Welch’s *t*-test (Independent Samples) comparisons of pre- and post-intervention outcomes. Statistical results included *p*-values (*p* < 0.05) to provide a thorough analysis of the findings.

## 3. Results

### 3.1. VR and RABT Balance Performance in Healthy Older Participants Based on Equivalent Baseline Ability

**Figure 2.**
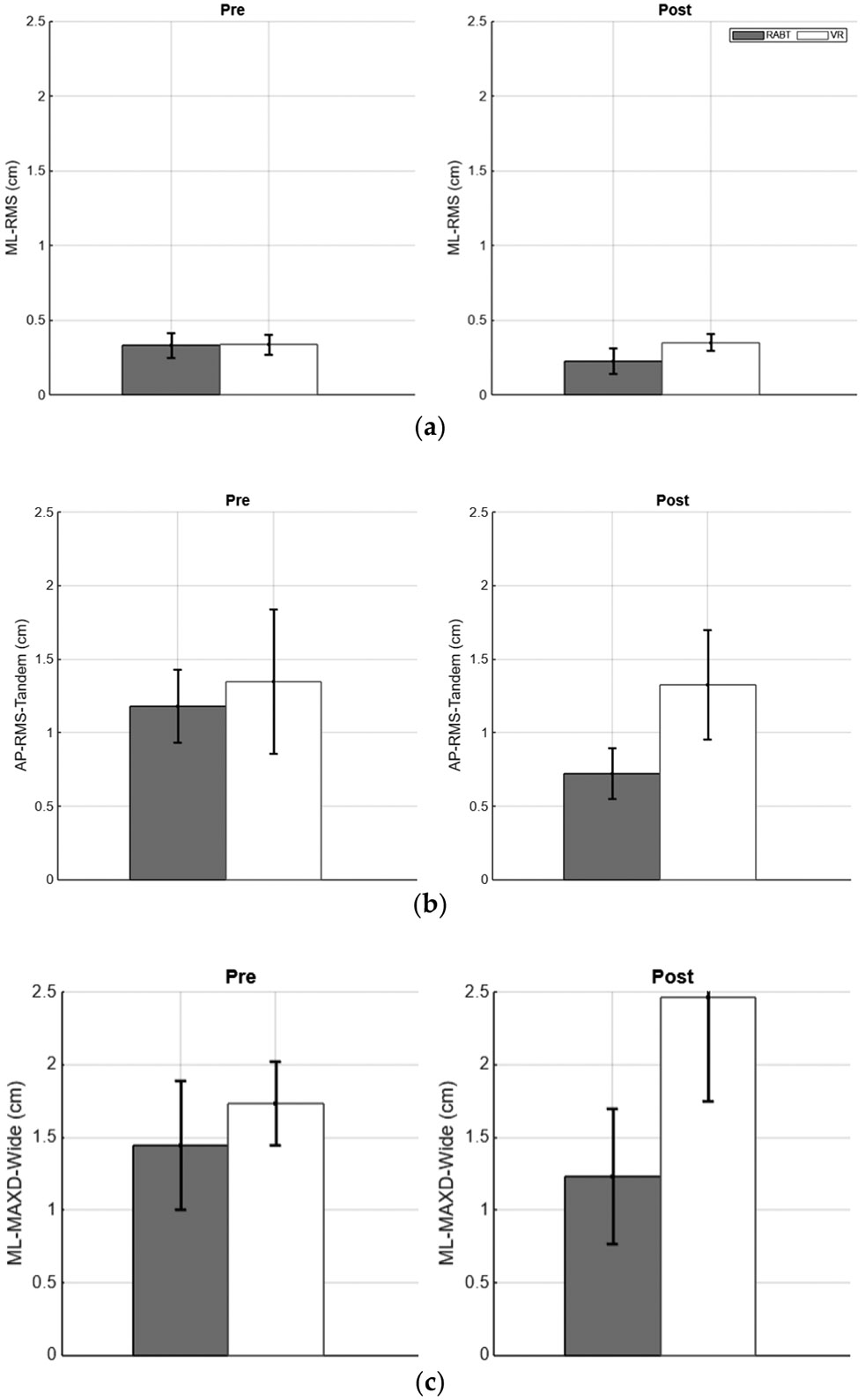
Pre-training (**left**) and post-training (**right**): (**a**) ML-RMS for wide stance, (**b**) AP-RMS for tandem stance (**middle**), and (**c**) ML-MAXD for wide stance values for RABT (gray) and VR (white) groups; standard error bars are shown.

**Figure 3.**
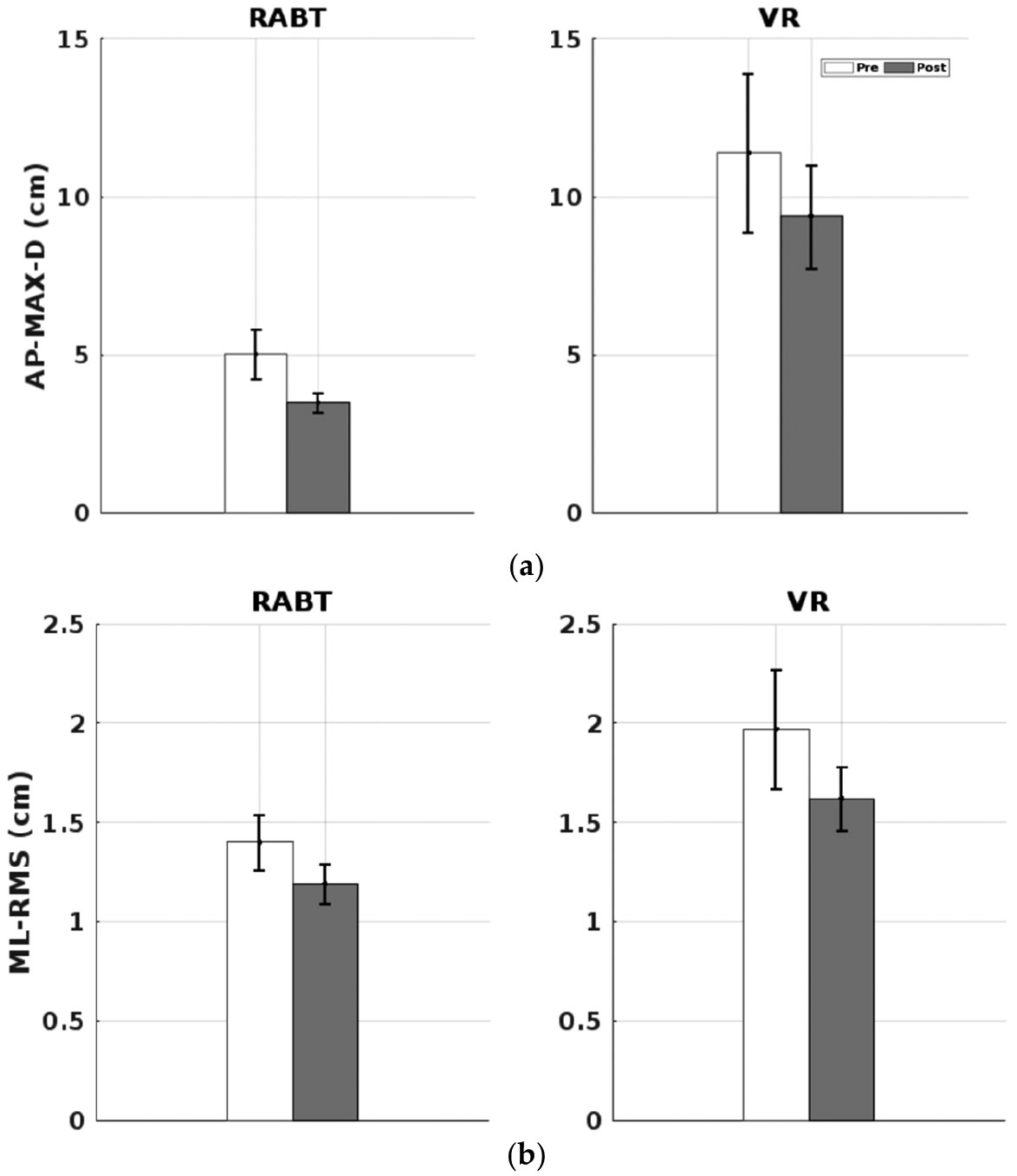
Pre-training (white) and post-training (gray) values for (**a**) AP-MaxD and (**b**) ML-RMS in tandem stance for RABT and VR groups. Error bars represent standard error.

**Figure 4.**
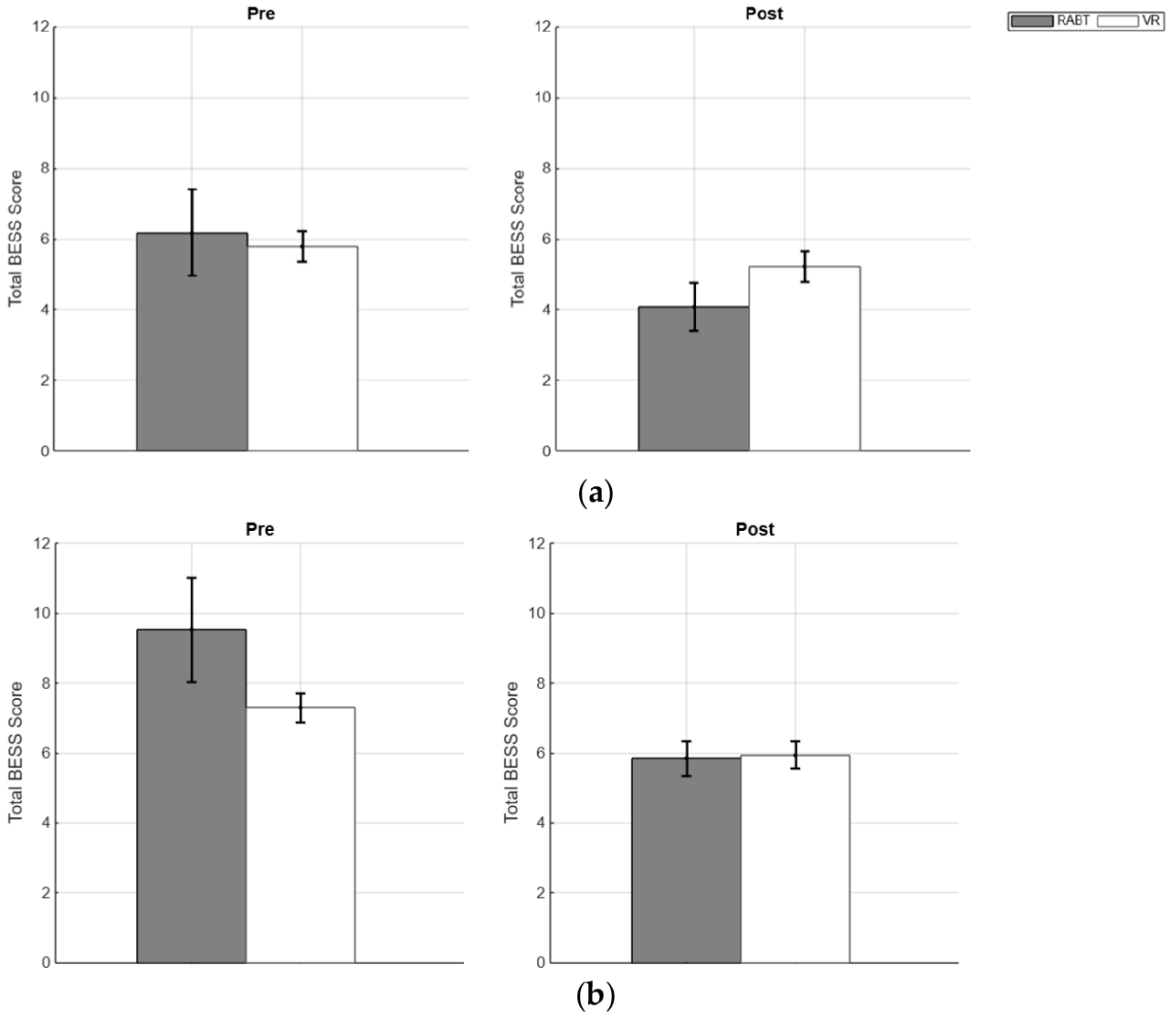
Pre-training (**left**) and post-training (**right**) BESS Scores for (**a**) firm and (**b**) foam RABT (gray) and VR groups (white) means, with standard error bars shown.

**Figure 5.**
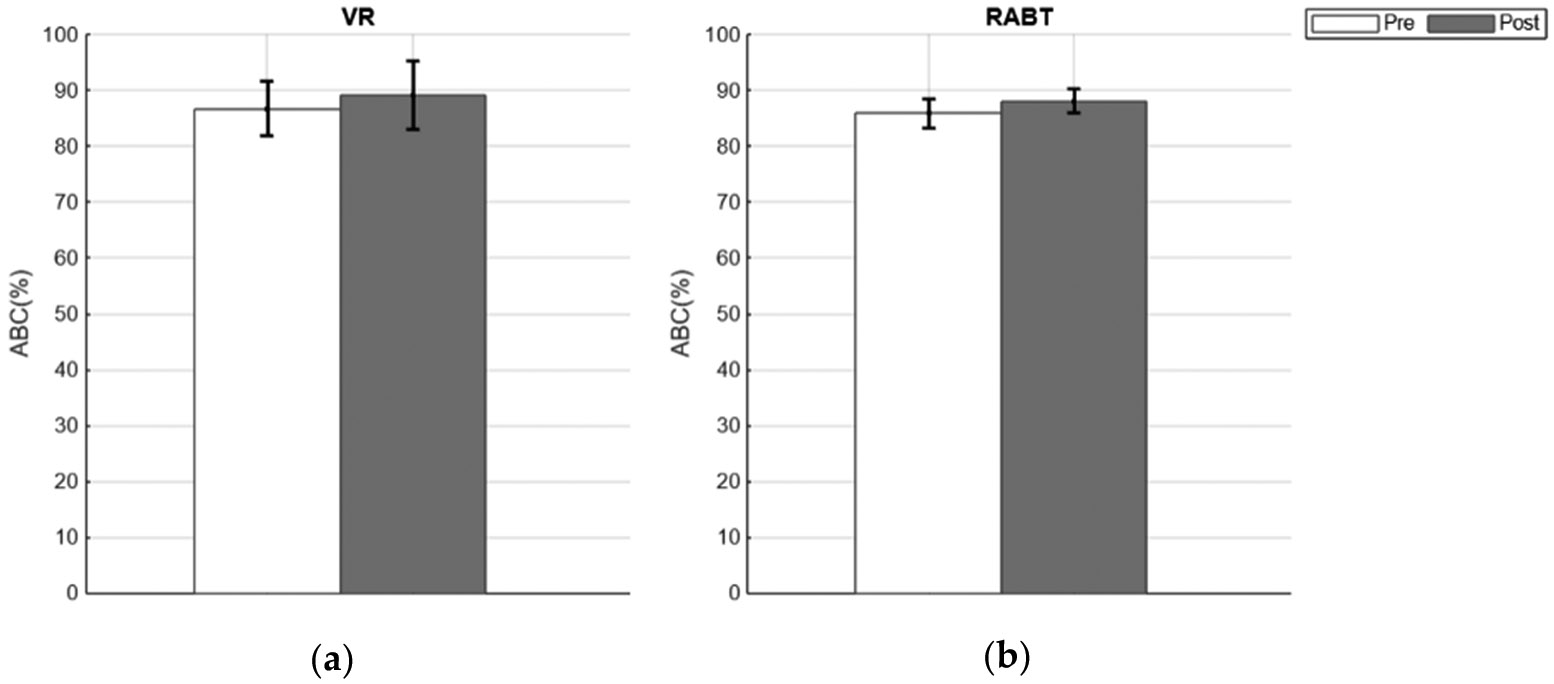
Comparison of Activities-Specific Balance Confidence (ABC) scores pre- (white) and post-training (gray) for (**a**) VR and (**b**) Robotic (healthy) group means with standard error bars shown.

**Figure 6.**
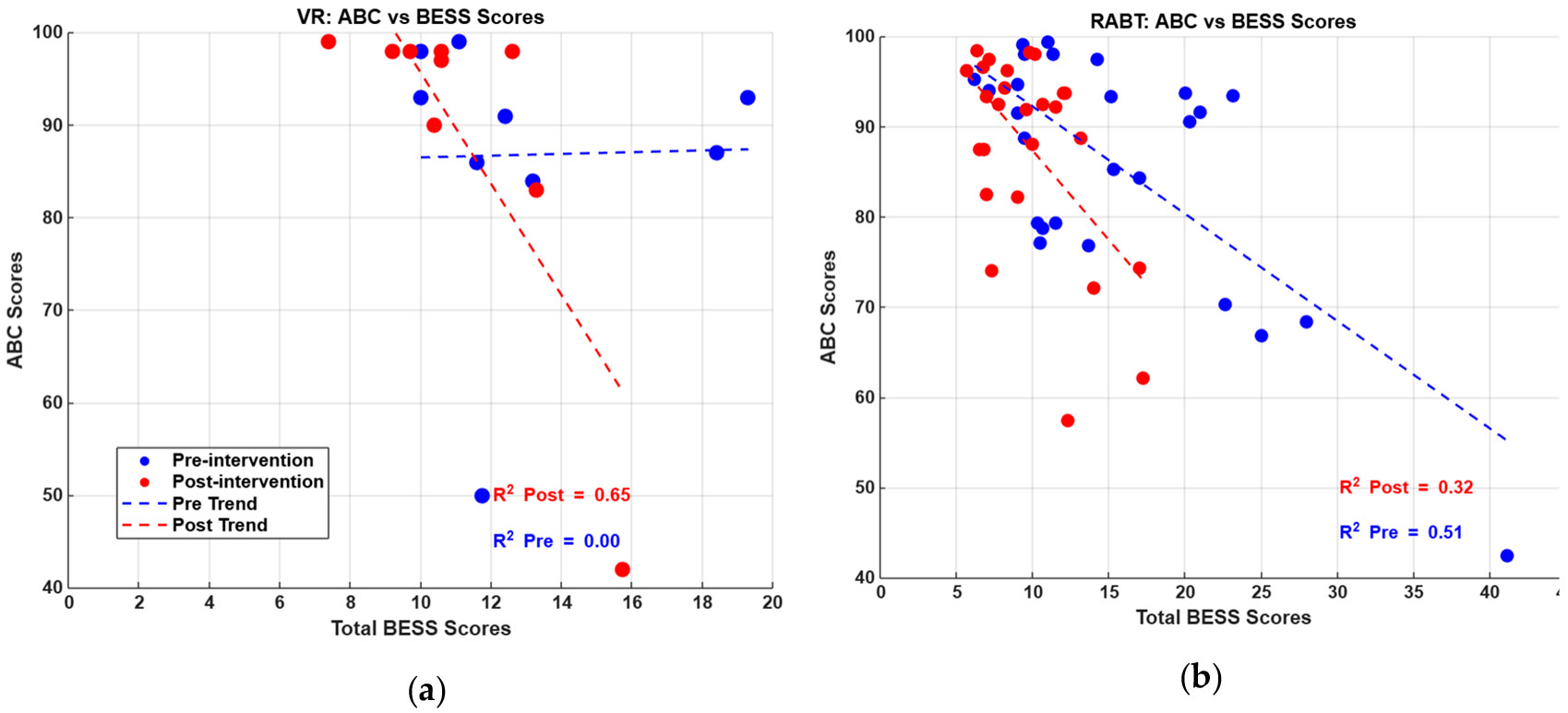
ABC scores versus total BESS Scores for (**a**) VR (**b**) RABT groups for pre-intervention (blue) and post-intervention (red); trend lines with corresponding R^2^ values are also shown.

The results below compare the average AP and ML COP parameters for all participants (i.e., RABT and VR RMS and MAXD (Figure 2) and for participants with insignificantly different pre-training scores (Figure 3), firm and foam BESS scores (Figure 4), ABC scores (Figure 5), and relationships between ABC (reflecting balance confidence) and total BESS scores (reflecting balance performance) (Figure 6).

Our examination focused on differential balance performance improvement according to participants’ baseline training score. Participants with lower baseline balance performance, as reflected by larger root mean square (RMS) and maximum displacement (MAXD) values in anteroposterior (AP) and mediolateral (ML) directions, demonstrated a greater potential for improvement compared to participants with higher baseline balance capacity.

To clarify these findings, we grouped participants into subgroups based on their pre-intervention balance performance scores. RABT and VR participants with insignificantly different pre-training (or baseline) scores were examined to determine their differences post-training. This allowed for controlled comparison of post-intervention effects between participants with comparable initial balance performance. There were no statistically significant differences across the investigated groups.

### 3.2. VR and RABT Balance Performance in Healthy Older Participants

Participant data were analyzed to assess changes in balance metrics following both interventions. Pre- and post-training averages for AP and ML directions (RMS and MaxD values) were calculated for healthy older adults in the RABT and VR training groups during wide and tandem stance conditions with eyes closed. The graphs below depict results that showed significant changes.

In the RABT, AP MaxD in tandem stance showed significant differences (*p* = 0.018). The VR group demonstrated significant changes in ML RMS values during tandem stance (*p* = 0.045).

No statistically significant differences were observed across the investigated groups. However, we noted trends in the BESS scores that may warrant further investigation with larger sample sizes. The RABT healthy group showed a mean reduction of 2.09 points on firm surfaces and 3.69 points on foam surfaces following robotic training; a reduction in score would mean an enhancement in balance performance on stable surfaces following robotic training.

The results from the ABC Scale assessments (Figure 5) reflected insignificant improvements in perceived balance confidence across both healthy groups. In the VR group, average balance confidence was approximately 87% (pre-training) to 89% (post-training), while the RABT showed similar results of 86% (pre-training) to 88% (post-training). These were insignificant changes.

As shown in Figure 6, prior to the intervention the VR group demonstrated a minimal correlation between balance confidence (ABC scores) and balance ability (BESS scores). The pre-intervention correlation coefficient (R^2^) was 0.00, indicating a complete absence of a linear relationship between participants’ perceived balance confidence and their actual balance performance. This suggests a significant disconnection between participants’ self-assessment of balance capabilities and their objective balance performance. The post-intervention analysis revealed a dramatic transformation in the relationship between balance confidence and balance performance. A pronounced negative correlation emerged, with the R^2^ increasing to 0.65. This significant shift demonstrates that the VR training substantially recalibrated participants’ perception of their balance abilities.

In contrast, the RABT group exhibited a moderate negative correlation between ABC and BESS scores before training, with an R^2^ of 0.51. This pre-existing relationship indicated that participants had a somewhat more accurate, albeit imperfect, perception of their balance abilities. As BESS scores increased (indicating more balance errors), ABC scores tended to decrease, reflecting a basic alignment between perceived and actual balance performance.

## 4. Discussion

For older adults, both lack of balance ability and lack of balance confidence are major challenges that contribute to falls and activity avoidance. The purpose of this study was to compare the effects of VR and RABT on balance ability and confidence in older adults. This topic is highly relevant because, while balance training can improve physical stability, if balance confidence remains unaddressed, individuals may still avoid daily activities due to fear of falling. Also, there is a question as to which training interventions are most effective within the older population that considers themselves as healthy, yet still have balance problems.

Our results demonstrated that participants who underwent RABT with the NaviGAITor system showed improvements in balance ability post-training as measured by COP and BESS metrics, though these changes were not statistically significant. In the second analysis, specific balance parameters showed improvement, particularly AP MaxD in tandem stance with significant difference (*p* = 0.018) in the RABT group and ML RMS values during tandem stance (*p* = 0.045) in the VR group.

The BESS results further supported these findings. The RABT group showed trends toward improvement, with a mean reduction of 2.09 points on firm surfaces and a more pronounced 3.69 points on foam surfaces. Intriguingly, greater improvements were observed on foam surfaces, which present a more challenging balance environment due to unreliable somatosensory information and increased demands on the vestibular system. This suggests that RABT may effectively enhance balance even under destabilizing conditions, though larger sample sizes would be needed to confirm statistical significance.

Balance confidence, as measured by the ABC scale, showed statistically insignificant changes for both groups. Both groups were overall healthy and had a relatively high perceived balance even prior to the interventions, thus changes post training were not substantial. However, VR training had a unique effect on participants’ self-awareness: post-training, a stronger negative correlation emerged between ABC scores and BESS errors (from R^2^ = 0.00 pre-training to R^2^ = 0.65 post-training), indicating that VR helped participants better align their perceived confidence with their actual balance ability. The analysis highlights the potential of VR interventions in not only improving balance performance but also in enhancing the relationship between participants’ self-awareness and perceptual accuracy of their physical capabilities. The RABT group, while showing some initial correlation (R^2^ = 0.51), did not demonstrate the same transformative effect observed in the VR intervention group.

These findings highlight the complementary strengths of VR and RABT. While RABT shows trends toward objective balance enhancement in specific parameters, VR appears to improve self-awareness and confidence calibration. A combined or alternating approach leveraging both technologies could offer a holistic strategy for fall prevention in older adults, addressing both physical stability and psychological barriers to mobility. Further, according to these observations, it may lead to training enhancements: RABT, typically used in and limited to a laboratory setting, and VR-based training, which would be more appropriate in one’s home setting, could both yield important improvements. Future studies with larger sample sizes would be valuable to further investigate the statistical significance of these observed trends.

## Data Availability

The data presented in this study are available on request from the corresponding author.
